# Colorectal cancer cell-derived CCL20 recruits regulatory T cells to promote chemoresistance via FOXO1/CEBPB/NF-κB signaling

**DOI:** 10.1186/s40425-019-0701-2

**Published:** 2019-08-08

**Authors:** Dan Wang, Li Yang, Weina Yu, Qian Wu, Jingyao Lian, Feng Li, Shasha Liu, Aitian Li, Zhiang He, Jinbo Liu, Zhenqiang Sun, Weitang Yuan, Yi Zhang

**Affiliations:** 1 Biotherapy Center, The First Affiliated Hospital of Zhengzhou University, Zhengzhou, Henan 450052 People’s Republic of China; 2 Cancer Center, The First Affiliated Hospital of Zhengzhou University, Zhengzhou, Henan 450052 People’s Republic of China; 3 Henan Key Laboratory for Tumor Immunology and Biotherapy, Zhengzhou, Henan 450052 People’s Republic of China; 4 Department of Anorectal Surgery, The First Affiliated Hospital of Zhengzhou University, Zhengzhou, Henan 450052 People’s Republic of China; 5 School of Life Sciences, Zhengzhou University, Zhengzhou, Henan 450001 People’s Republic of China

**Keywords:** Chemoresistance, CCL20, FOXO1/CEBPB/NF-κB, Regulatory T cells, Colorectal cancer (CRC)

## Abstract

**Background:**

Colorectal cancer (CRC) is one of the most common forms of cancer worldwide. The tumor microenvironment plays a key role in promoting the occurrence of chemoresistance in solid cancers. Effective targets to overcome resistance are necessary to improve the survival and prognosis of CRC patients. This study aimed to evaluate the molecular mechanisms of the tumor microenvironment that might be involved in chemoresistance in patients with CRC.

**Methods:**

We evaluated the effects of CCL20 on chemoresistance of CRC by recruitment of regulatory T cells (Tregs) in vitro and in vivo.

**Results:**

We found that the level of CCL20 derived from tumor cells was significantly higher in Folfox-resistant patients than in Folfox-sensitive patients. The high level of CCL20 was closely associated with chemoresistance and poor survival in CRC patients. Among the drugs in Folfox chemotherapy, we confirmed that 5-FU increased the expression of CCL20 in CRC. Moreover, CCL20 derived from 5-FU-resistant CRC cells promoted recruitment of Tregs. Tregs further enhanced the chemoresistance of CRC cells to 5-FU. FOXO1/CEBPB/NF-κB signaling was activated in CRC cells after 5-FU treatment and was required for CCL20 upregulation mediated by 5-FU. Furthermore, CCL20 blockade suppressed tumor progression and restored 5-FU sensitivity in CRC. Lastly, the expression of these signaling molecules mediating chemoresistance was closely correlated with poor survival of CRC patients.

**Conclusions:**

CRC cell-secreted CCL20 can recruit Tregs to promote chemoresistance via FOXO1/CEBPB/NF-κB signaling, indicating that the FOXO1/CEBPB/NF-κB/CCL20 axis might provide a promising target for CRC treatment.

**Electronic supplementary material:**

The online version of this article (10.1186/s40425-019-0701-2) contains supplementary material, which is available to authorized users.

## Introduction

Colorectal cancer (CRC) is one of the most common forms of cancer worldwide [1]. Recurrence, metastasis, and drug resistance in the course of chemotherapy pose a great threat to CRC patients [2], especially as chemoresistance limits the effectiveness of chemotherapeutic agents to a large extent [3]. Although the mechanisms of anticancer drug resistance have been broadly investigated, they are not completely understood.

Recently, it is becoming increasingly apparent that the tumor microenvironment plays a crucial role in promoting tumor resistance to chemotherapy in solid cancers [4, 5]. Therefore, effective targets to overcome resistance are necessary to improve the survival and prognosis of tumor patients.

Many factors including immunosuppressive cells, cytokines and chemokines contribute to drug resistance in the tumor microenvironment [6, 7]. Higher infiltration of regulatory T cells (Tregs) could be significantly correlated with resistance to antiangiogenic therapy in metastatic renal cell carcinoma [8]. Inducible nitric oxide synthase derived from tumor-associated macrophages protects glioma cells from chemotherapeutic drug-induced apoptosis [9]. Furthermore, CXCL12 or stromal cell-derived factor 1 is considered one of the most significant chemokines to promote drug resistance in various cancers [10–12]. Anti-apoptotic molecules such as IL-6, IL-10 and TNFα are implicated in drug resistance in non-Hodgkin’s lymphoma, breast cancer, and glioma [13–16]. Our previous study demonstrated the important role of CXCR7 in the control of chemoresistance induced by IL-6 in esophageal squamous cell carcinoma [17].

Therefore, the molecular mechanisms underlying the regulation of drug resistance by the tumor microenvironment could provide potential targets to overcome the chemoresistance of CRC. In this study, we found that colorectal cancer cell-derived chemokine (C-C motif) ligand 20 (CCL20) induced recruitment of Tregs via FOXO1/CEBPB/NF-κB signaling, and that Tregs further promoted chemoresistance of CRC. This study demonstrated the important role of CCL20 in regulating chemoresistance induced by FOXO1/CEBPB/NF-κB signaling in CRC. Thus, the FOXO1/CEBPB/NF-κB/CCL20 axis might provide a potential molecular target for CRC therapy.

## Materials and methods

### Patients and tumor samples

Serum samples from 87 CRC patients who underwent traditional chemotherapy (Folfox therapy), 55 tumor tissues from CRC patients who underwent neoadjuvant chemotherapy (Folfox therapy), and 104 tumor tissues from CRC patients who did not undergo chemotherapy were obtained from The First Affiliated Hospital of Zhengzhou University from the year 2011 to 2015. Patients were divided into two groups according to the RECIST 1.1 criteria as sensitive patients including ‘Complete Response,’ ‘Partial Response,’ and ‘Stable Disease’, and resistant patients including ‘Progressive Disease’. The patients were staged according to the UICC-TNM classification and all the samples were confirmed by pathological analysis. These patients were subjected to diagnosis using conventional histology. The clinical data of the patients are shown in Table 1. All patients signed written informed consent in accordance with the standards defined by the Institutional Review Board of the hospital (Ethics approval number: Science-2010-LW-1213).

**Table 1:** Characteristics of CRC patients

Characteristics	CRC patients with traditional Folfox		CRC patients with neoadjuvant Folfox		CRC patients without Folfox	
	Number	%	Number	%	Number	%
Gender						
Male	46	52.8	31	56.4	51	49.0
Female	41	47.1	24	23.1	53	51.0
Age (years)						
< 60	44	50.6	29	52.7	57	54.8
≥ 60	43	49.4	26	47.3	47	45.2
Location						
Colon	56	64.4	36	65.5	61	58.7
Rectum	31	35.6	19	34.5	43	41.3
Tumor size						
< 40 mm	50	57.5	33	60.0	60	57.7
≥ 40 mm	37	42.5	22	40.0	44	42.3
Pathological type						
Adenocarcinoma	77	88.5	45	81.8	94	90.3
Others	10	11.5	10	18.2	10	9.6
Lymph node metastasis						
Yes	41	47.1	25	45.5	53	51.0
No	46	52.9	30	54.5	51	49.0
Distant metastasis						
Yes	34	39.1	27	49.0	23	22.1
No	53	60.9	28	50.9	81	77.9
TNM Stage						
I	5	5.7	5	9.0	12	11.5
II	36	41.4	18	32.7	39	37.5
III	25	28.7	6	10.9	30	28.8
IV	21	24.1	27	49.0	23	22.1
Differentiation						
Low	5	5.7	7	12.7	6	5.7
Low-moderate	15	17.2	10	18.2	18	17.3
Moderate	67	77.0	38	69.1	80	74.8

### Multiplex assay

A multiplex assay was carried out to identify which factor plays a key role in determining and maintaining the chemoresistant properties of CRC cells. The levels of cytokines and chemokines in the serum of chemoresistant and chemosensitive CRC patients were analyzed using a multi-analyte flow assay kit (Biolegend, USA) including 13 human cytokines and 13 human chemokines, according to the manufacturer’s instructions.

### ELISA

The CCL20 concentration in the serum of chemoresistant and chemosensitive CRC patients, and in the supernatants of different conditional CRC cell lines was measured by ELISA (R&D Systems Inc., USA) as described previously [18].

### Immunohistochemistry and immunofluorescence staining

The protocols used for immunohistochemistry and immunofluorescence are described elsewhere [18]. Anti-CCL20, anti-CD326, anti-FOXP3 (1:300; Abcam, USA), anti-P-P65, anti-FOXO1, and anti-CEBPB (1:300; Cell Signaling Technology, USA) were used as primary antibodies. For immunohistochemistry, three fields of view per sample were imaged. The intensity of immunostaining was considered when analyzing the data. The percentage scoring of immunoreactive tumor cells was as follows: 0 (< 10%), 1 (10–40%), 2 (40–70%), and 3 (> 70%). Staining intensity was visually scored and stratified as follows: 0 (negative), 1 (yellowish), 2 (light brown), and 3 (dark brown). Immunoreactivity scores (IRS) were obtained by multiplying the two items to a total score and ranged from 0 to 9. Protein expression levels were further analyzed by classifying IRS values as low (based on an IRS value ≤5) and as high (based on an IRS value > 5). For immunofluorescence, the sections were treated with 1% Triton X100 in 0.01 M PBS. Cy3- and FITC-conjugated secondary antibodies (1:500; BioLegend, USA) were used to detect the primary antibodies. Nuclear staining was performed with DAPI (11,000; Solarbio, China). The samples were visualized using a fluorescence microscope (Olympus, IX71, Japan).

### RNA extraction and qPCR

Total RNA was extracted from cells and tissues with TRIzol reagent (Invitrogen Corporation, USA) according to the manufacturer’s instructions. The concentration and purity of RNA were detected using Nano Drop 2000 (Thermo Scientific, USA). First-strand cDNA was synthesized from 1 μg of total RNA using the Prime Script RT reagent Kit With gDNA Eraser (TaKaRa, Japan). qPCR was performed using SYBR Premix Ex Taq II (TaKaRa, Japan) on AgilentMx3005P. GAPDH was used as an endogenous control for normalization.

### Isolation of lymphocytes

Human CD4 magnetic beads (Miltenyi Biotec) were used for the isolation of CD4^+^ T cells from peripheral blood mononuclear cells (PBMCs) according to the manufacturer’s instructions. CD4^+^CD25^+^ Tregs [19] and CD4^+^CD25^−^ cells were sorted from the PBMCs of healthy donors and CRC patients using the MoFlo XDP cytometer (Beckman Coulter). The positive rate of cells after purification was more than 90%.

### Flow cytometric evaluation of apoptosis

Cells were harvested and washed twice with ice-cold PBS. The cells were then suspended in Annexin V-binding buffer to a final concentration of 10^6^ cells/ml. Thereafter, cells were incubated with AlexaFluor 647 Annexin V (Biolegend, USA) for 15 min at 4 °C in the dark, and PI (Sigma, USA) was added. Samples were immediately analyzed by flow cytometry (FACSCanto II, BD, USA).

### Migration assay

A 5-μm pore diameter chamber (Corning, USA) was used in a Transwell assay, wherein 1 × 10^5^ purified CD4^+^CD25^+^ Tregs from the peripheral blood mononuclear cells (PBMCs) of healthy donors were seeded in the upper chamber, and 600 μL of SW620 cell supernatant was co-cultured in the bottom chamber. Recombinant human CCL20 (10 ng/ml; Peprotech, USA) and anti-CCL20 antibody (15 ng/ml; Abcam, USA) were added to these cells. The cells were incubated at 37 °C with 5% CO_2_ for 48 h.

In another migration assay, 1 × 10^6^ PBMCs or 1 × 10^5^ Tregs were seeded in the upper chamber with 5-μm diameter pores (Corning, USA). Then, 600 μL of SW620 cell supernatant treated with 5-FU (10 μg/ml; Sigma, USA) for 48 h was co-cultured in the bottom chamber. Anti-CCL20 antibody (15 ng/ml) or QNZ (10 nM; Selleck, China) was added to these cells. After incubation for 24 h, the migrated cells were stained with 0.1% crystal violet and counted. All experiments were repeated three times independently.

### Cell viability assay

Cell proliferation rate was determined using the CCK assay (Dojindo, Japan) according to the manufacturer’s protocol. Cells were seeded in 5 replicates in a 96-well plate at a density of 5000 cells per well and were cultured with 100 μL DMEM containing 10% FBS. Cells were incubated with 10 μL of CCK-8 for 4 h at 37 °C. Cell viability was determined every day by measuring the absorbance at 450 nm with a plate reader (MULTISKANMK3, Thermo Scientific, USA).

### Dual-luciferase reporter assay

SW620 cells were cultured at a concentration of 3000 cells per well in 96-well plates. After 24 h, the cells were transfected with the expression vector (0.01 μg/well, CCL20 construct) and 0.5 μg of the reporter plus the pcDNA3.1 expression vector. The PRL-TK vector constitutively expresses Renilla luciferase and thus served as an indicator for estimating the transfection efficiency. Luciferase assays were conducted according to the manufacturer’s instructions using a Dual Luciferase Reporter System (Promega Benelux, Leiden, Netherlands) to measure luciferase activity, measured with a Lumimark luminometer (Bio-Rad Laboratories, Hercules, CA, USA).

### Lentiviral generation and cell sorting

SW620 cells were stably transfected with a vector containing a FOXO1-specific small hairpin RNA (shRNA) or CEBPB-specific shRNA to knockdown FOXO1 or CEBPB expression. All inserted sequences were confirmed by DNA sequencing. After vector transfection, the transfected cells were sorted by flow cytometry (Beckman MoFlo XDP, USA) according to the expression of green fluorescent protein (GFP). The decreased expression of FOXO1 or CEBPB in SW620 cells was confirmed by qRT-PCR.

### Western blotting analysis

Cells were extracted into cold lysis buffer containing 50 mM Tris-HCl (pH 7.5), 150 mM NaCl, 1 mM EDTA, 1 mM MgCl_2_, 0.5% Triton X-100, phosphatase inhibitor mix, and protease inhibitor mix. Protein concentration was determined using the BCA method (Biyuntian, China). The following primary antibodies were used: anti-FOXO1, anti-CEBPB, anti-phospho-P65, and anti-β-actin (1:1000; Cell Signaling Technology, USA) as the control. These primary antibodies were detected with a goat polyclonal secondary antibody to rat (1:1000; BioLegend, USA). The band images were digitally captured and quantified with a Fluor Chem FC2 imaging system (Alpha Innotech, USA).

### Animal model

In one set of experiments, 10 female NOD SCID mice (Beijing Vital River Laboratory Animal Technology Co. Ltd., China) aged 6 weeks were randomly divided into two groups (five mice/group). Both groups received hypodermic injections of 5 × 10^6^ HCT116 cells (D-7). Mice were inspected and tumor growth was evaluated by measuring the length and width of the tumor mass using calipers. When tumor volumes reached 250 mm^3^ (D0), 5-FU (10 mg/kg/day, i.p.; Sigma, USA) treatment was started. Two days before the mice were sacrificed, CD4^+^ cells (5 × 10^6^ cells) from peripheral blood in CRC patients were transplanted through the caudal vein (D2).

In the CCL20 blockade assay, 5 × 10^6^ HCT116 cells were injected subcutaneously into the mice (D-7). Seven days after cell implantation, anti-CCL20 antibody (1 mg/kg; Abcam, USA) or DMSO as a control was administered locally to the mice every 2 days for 2 weeks (D0, 2, 4, 6, 8, 10, 12). At day 6–12 after anti-CCL20 antibody administration, mice were subjected to 5-FU (10 mg/kg/day, i.p.) treatment. At day 14 after anti-CCL20 antibody usage, CD4^+^CD25^+^ Tregs (5 × 10^6^ cells) from peripheral blood in CRC patients were transplanted through the caudal vein (D14). Seventeen days later, the mice were sacrificed by cervical dislocation and the tumors were isolated for further analysis. All animal procedures were conducted in accordance with the Guide for the Care and Use of Laboratory Animals and were approved by the Institutional Animal Care and Use committee of the First Affiliated Hospital of Zhengzhou University.

### Public clinical datasets

We obtained the raw gene expression of 640 CRC cases in The Cancer Genome Atlas (TCGA) using cBioPortal for Cancer Genomics (www.cbioportal.org) to evaluate the correlation between FOXP3 and BCL2, WNT1, ATP8A2, and VIM expression. In addition, correlations in FOXO1, CEBPB, RELA (P65), and FOXP3 expression levels were determined using Pearson correlation coefficients.

### Statistical analysis

Data of different groups were compared using Student’s *t*-test, chi-squared test, or one-way ANOVA. Overall survival curves were plotted according to the Kaplan-Meier method. Spearman correlation analysis was also performed. Statistical analyses were performed using Graph Pad Prism 5 software (Graph Pad Software, La Jolla, CA, USA). *P* < 0.05 was considered to indicate a statistically significant difference.

## Results

### CCL20 levels are increased in chemoresistant CRC patients

**Fig. 1:**
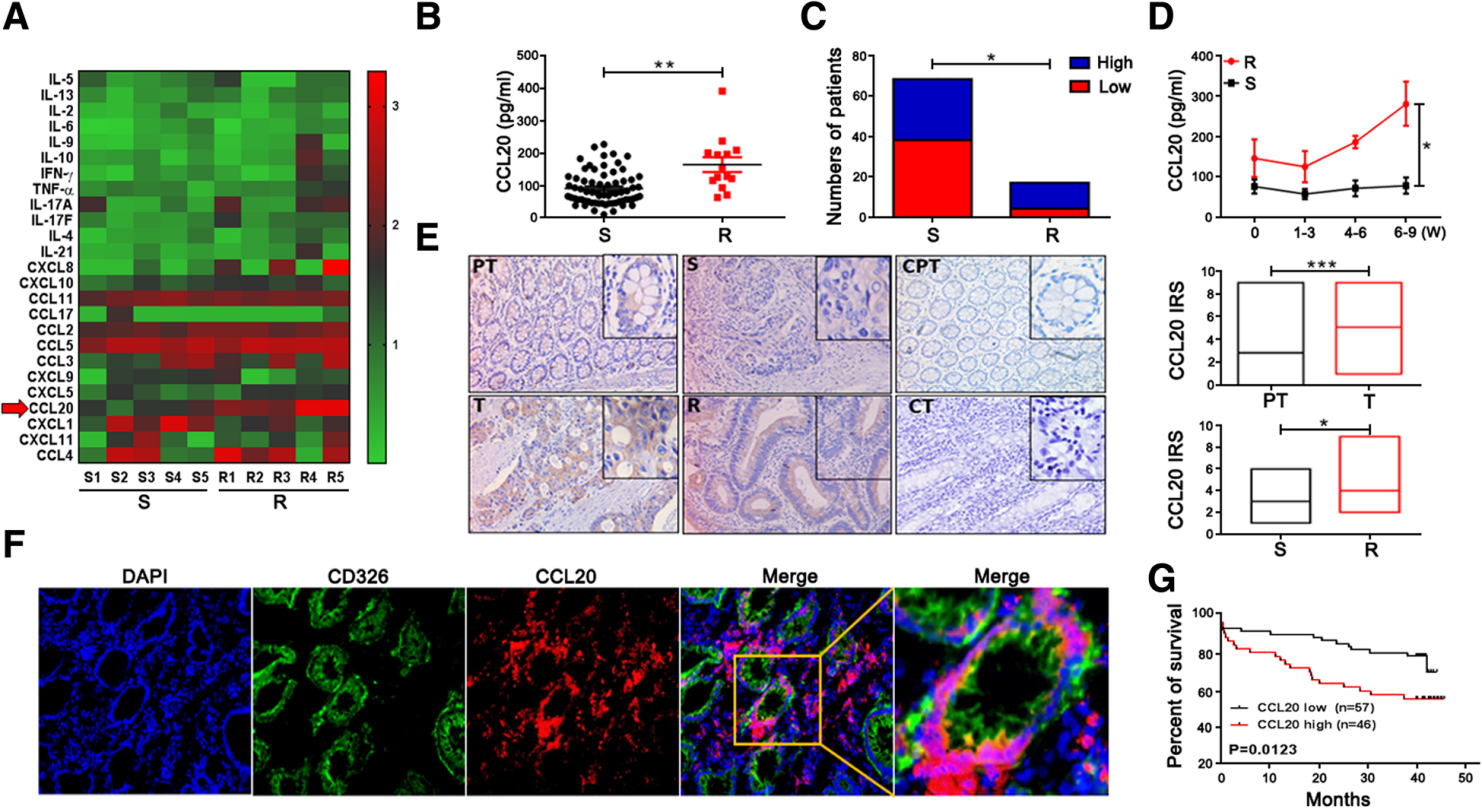
CCL20 levels are elevated in chemoresistant CRC patients. **a** Heatmap showing the concentration of 13 cytokines and 13 chemokines (pg/mL) in serum samples from CRC patients with chemosensitivity (S) and chemoresistance (R) as measured by multiplex assay. **b** CCL20 concentration (pg/ml) in serum samples from CRC patients (*n* = 87) with chemosensitivity and chemoresistance as measured by ELISA. **c** Histogram showing the percentage of chemosensitivity and chemoresistant patients with high and low levels of CCL20. The 87 serum samples from CRC patients were stratified as ‘high’ and ‘low’ according to the median CCL20 level (median = 81.55 pg/ml). **d** Concentration of CCL20 (pg/ml) in the serum obtained from chemosensitive and chemoresistant CRC patients at different chemotherapy stages as measured by ELISA. **e** Immune reactivity score (IRS) of CCL20 in peritumor and tumor tissues with neoadjuvant chemotherapy (*n* = 55) or without neoadjuvant chemotherapy (*n* = 104) as evaluated by immunohistochemistry (200 ×). PT- paired peritumor tissues, T-tumor tissues, S-tumor tissues with chemo-sensitivity, R-tumor tissues with chemoresistance, CPT-negative staining for paired peritumor tissues, CT-negative staining for tumor tissues. **f** Colorectal tumor tissues subjected to double immunofluorescence for CD326 (green), CCL20 (red), and DAPI (blue). One representative micrograph is shown (200 ×). **g** Kaplan-Meier survival curves for 104 CRC patients without neoadjuvant chemotherapy. The samples were stratified as ‘high’ and ‘low’ according to the IRS of CCL20 (IRS = 5). *P < 0.05, **P < 0.01, ***P < 0.001

To determine the key immune-related factors that play induce chemoresistance in CRC patients, the expression levels of multiple chemokines and cytokines in the serum of Folfox-sensitive and Folfox-resistant patients were detected by multiplex assay. We found that the level of CCL20 was significantly higher in the serum of Folfox-resistant patients than in the serum of Folfox-sensitive patients (Fig. 1a, Additional file 1: Figure S1). For confirmation, we further determined the protein expression of CCL20 in the serum of CRC patients by ELISA. Similarly, the protein level of CCL20 in the serum of Folfox-resistant patients was significantly higher than that in the serum of Folfox-sensitive patients (Fig. 1b). The percentage of Folfox-resistant patients with a high level of CCL20 in all Folfox-resistant patients was significantly increased compared to that in Folfox-sensitive patients (Fig. 1c). Moreover, the CCL20 level in the serum of Folfox-resistant patients was significantly higher than that in the serum of Folfox-sensitive patients in a time-dependent fashion (Fig. 1d). Immunohistochemistry results showed that CCL20 expression in tumor tissues was obviously higher than that in peritumor tissues (*P* < 0.001, Fig. 1e), and similarly higher levels were observed in tumor tissues from Folfox-resistant patients who received neoadjuvant chemotherapy (Fig. 1e). To identify whether CCL20 was produced by colorectal cancer cells, immunofluorescence was performed to co-stain CCL20 and CD326 in tumor tissues. The result showed that CCL20 was indeed derived from CD326^+^ cancer cells (Fig. 1f). In addition, the patients with low levels of CCL20 showed good overall survival (Fig. 1g). Therefore, these results suggest that the level of CCL20 is increased in chemoresistant CRC patients, and that CCL20 is a prognostic indicator in CRC patients.

### 5-FU increases the expression of CCL20 in CRC

**Fig. 2:**
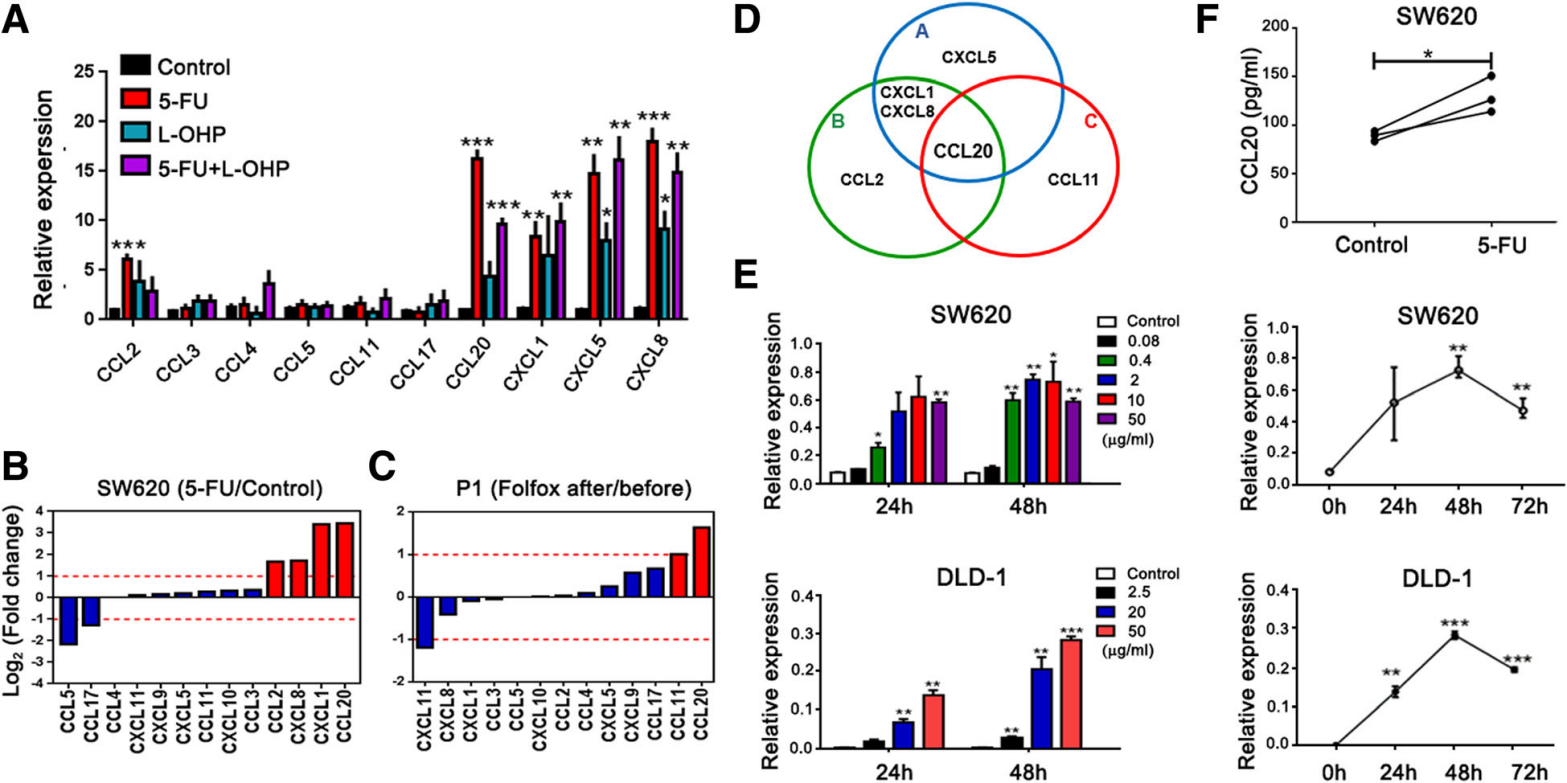
5-FU increases the expression of CCL20 in CRC. **a** Relative expression of related chemokines in SW620 cells treated with chemotherapy [5-FU (2 μg/ml), L-OHP (10 μg/ml), 5-FU (2 μg/ml) + L-OHP (10 μg/ml)] was analyzed by qPCR. **b** Log_2_ fold changes in related chemokine expression of SW620 cells treated with 5-FU compared to the control using a multiplex assay. The supernatant was pooled from three experiments. **c** Log_2_ fold changes in related chemokine expression of tumor tissues using multiplex assay for one CRC patient before and after Folfox treatment. The serum was pooled from three patients. **d** Graph based on the above results (A-C), showing that CCL20 was the key and dominant factor in the altered chemokine expression before and after chemotherapy, especially after 5-FU treatment. A-results from **a**, B-results from **b**, C-results from **c**. **e** Relative expression of CCL20 in SW620 and DLD-1 cells treated with different doses of 5-FU (μg/ml) at different timepoints (left) was analyzed by qPCR. Relative expression of CCL20 in SW620 and DLD-1 cells treated with the 5-FU concentration at which the most significant change occurs (2 μg/ml for SW620, 50 μg/ml for DLD-1) at different timepoints (right). **f** Concentration of CCL20 (pg/ml) in supernatants of SW620 cells with or without 5-FU (10 μg/ml) treatment was measured by ELISA. *P < 0.05, **P < 0.01, ***P < 0.001

To identify which drug in Folfox chemotherapy was responsible for inducing the high level of CCL20 in CRC, we investigated the effect of different drugs in Folfox (5-FU, L-OHP, 5-FU + L-OHP) on the changes in chemokine expression. We found that CCL20 expression was significantly increased by 5-FU alone than by L-OHP alone or by the combination therapy of 5-FU and L-OHP (Fig. 2a). Multiplex assay results showed that 5-FU remarkably enhanced CCL20 expression in SW620 cells compared to the control (Fig. 2b). Clinically, after treatment with Folfox, CCL20 expression in the serum of CRC patients was obviously increased (Fig. 2c). Based on the above results, CCL20 was found to be the key dominant factor in the changes in chemokine expression before and after chemotherapy, especially 5-FU treatment (Fig. 2d). To further evaluate the effect of 5-FU on CCL20 expression in cancer cells, we determined the mRNA expression of CCL20 in SW620 and DLD-1 cells after treatment with 5-FU, and found that CCL20 was significantly increased especially at 48 h, in a dose-dependent manner (Fig. 2e). Furthermore, the level of CCL20 in the supernatant of SW620 cells was obviously increased after treatment with 5-FU in vitro (Fig. 2f). Taken together, these results indicate that 5-FU increases the expression of CCL20 in CRC.

### 5-FU-resistant CRC cell-derived CCL20 promotes the recruitment of Tregs

**Fig. 3:**
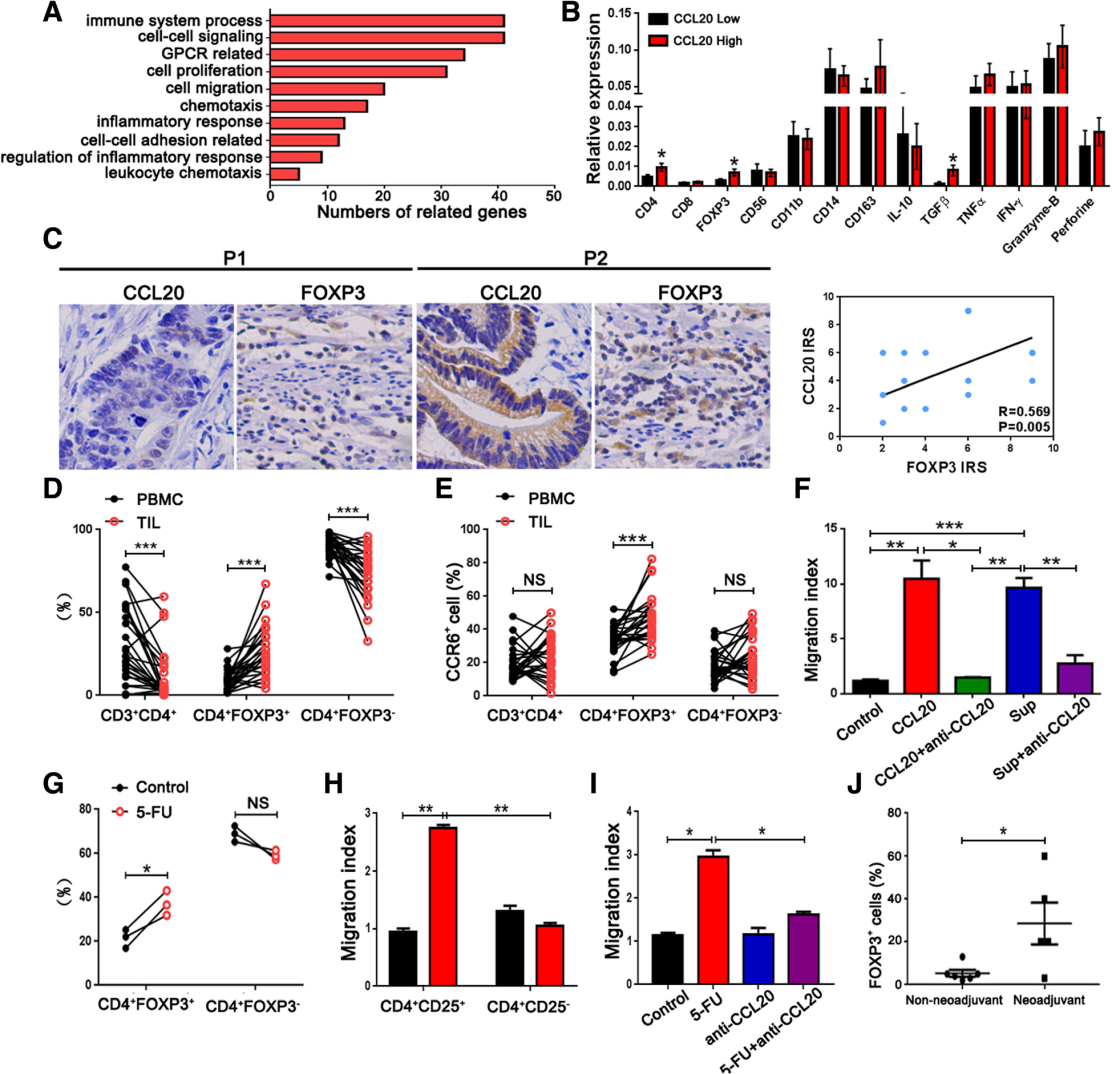
5-FU-resistant CRC cell-derived CCL20 promotes recruitment of Tregs. **a** GO analysis showed the top 10 gene functions that were mostly different among the CCL20 high and low expression based on TCGA data. **b** Relative expression of immune system-related genes in tumor tissues from 104 CRC patients with high and low CCL20 expression, stratified by the median, was analyzed by qPCR. **c** The relationship between the expression of CCL20 and FOXP3 in CRC tissues was detected by immunohistochemistry (200 ×; P1: low expression, P2: high expression). **d** The percentage of CD3^+^CD4^+^, CD4^+^FOXP3^+^ and CD4^+^FOXP3^−^ cells from TILs and paired PBMCs of 26 CRC patients was analyzed by flow cytometry. **e** The percentage of CCR6^+^ cells in CD3^+^CD4^+^, CD4^+^FOXP3^+^, and CD4^+^FOXP3^−^ cells from TILs and PBMCs of 26 CRC patients was analyzed by flow cytometry. **f** Migration of purified CD4^+^CD25^+^ Tregs from healthy donors co-cultured with recombinant human CCL20 or the supernatants of SW620 cells before and after treatment with CCL20 inhibitor was analyzed by Transwell assay. The migration index was calculated by dividing the number of cells that migrated in the indicated groups by the number migrating in control groups (*n* = 3). **g** Migration of purified CD4^+^ cells from healthy donors co-cultured with the supernatants of SW620 cells before and after treatment with 5-FU was analyzed by Transwell assay. **h** Migration of purified CD4^+^CD25^+^ Tregs and CD4^+^CD25^−^ cells from healthy donors co-cultured with the supernatants of SW620 cells treated with 5-FU was analyzed by Transwell assay (*n* = 3). **i** Migration of SW620 cells co-cultured with 5-FU and/or anti-CCL20 antibody was analyzed by Transwell assay (*n* = 3). **j** The percentage of FOXP3^+^ cells in the tumor tissues of CRC patients (*n* = 12) with or without neoadjuvant therapy was detected by immunohistochemistry. *P < 0.05, **P < 0.01, ***P < 0.001, NS- non-significant

To further evaluate the effect of CCL20, we analyzed the different functions of CCL20-high or -low expression from TCGA data using Gene Ontology (GO) analysis. The results revealed that the most differentially expressed genes were related to immune system processes (Fig. 3a). Next, to investigate the role of CCL20 in remodeling the tumor microenvironment, we analyzed the immune-related gene expression in tumor tissues with high and low CCL20 expression, and found that the expression of Foxp3, CD4, and TGF-β, one of the mainly functional molecules secreted from Tregs [20], in tumor tissues with high CCL20 expression was significantly higher than that in tumor tissues with low CCL20 expression (Fig. 3b). CCL20 expression was positively associated with FOXP3 expression in tumor tissues as analyzed by immunohistochemistry (Fig. 3c). The percentage of CD4^+^FOXP3^+^ Treg cells [21] from the tumor infiltrating lymphocytes (TILs) of CRC patients was obviously higher than that from paired PBMCs (Fig. 3d). Meanwhile, the percentage of CCR6^+^ cells (CCR6, the receptor of CCL20) in CD4^+^FOXP3^+^ cells from TILs was obviously higher than that from PBMCs (Fig. 3e). The enrichment of CCR6^+^CD4^+^FOXP3^+^ cells in TILs indicated the CCL20-driven migration of Treg lymphocytes in CRC patients. Transwell assay results showed that recombinant human CCL20 and the supernatants of SW620 cells promoted the migration of purified CD4^+^CD25^+^ cells from healthy donors, which could be inhibited using anti-CCL20 antibody (Fig. 3f). These data suggest that CCL20 can recruit and promote Treg infiltration in colorectal tumor tissues.

Next, we further investigated whether 5-FU-mediated CCL20 upregulation could affect Treg recruitment. After treatment with 5-FU, SW620 cell-derived supernatant was added to CD4^+^ cells in a Transwell assay. The frequency of CD4^+^FOXP3^+^ cell migration was increased compared to that in the control, whereas the CD4^+^FOXP3^−^ cell migration frequency was decreased (Fig. 3g). Moreover, the supernatants of SW620 cells treated with 5-FU promoted the migration of purified CD4^+^CD25^+^ cells and showed no significant difference in purified CD4^+^CD25^−^ cells (Fig. 3h). After treatment with anti-CCL20 antibody, the migration ability of SW620 cells treated with 5-FU was significantly decreased (Fig. 3i). In addition, immunohistochemistry results showed that FOXP3 expression in the tumor tissues of CRC patients with neoadjuvant therapy (Folfox therapy) was higher than that without neoadjuvant therapy (Fig. 3j). All of these data demonstrate that CCL20 derived from 5-FU-resistant CRC cells promotes the recruitment of Tregs.

### Tregs enhance the chemoresistance of CRC to 5-FU

**Fig. 4:**
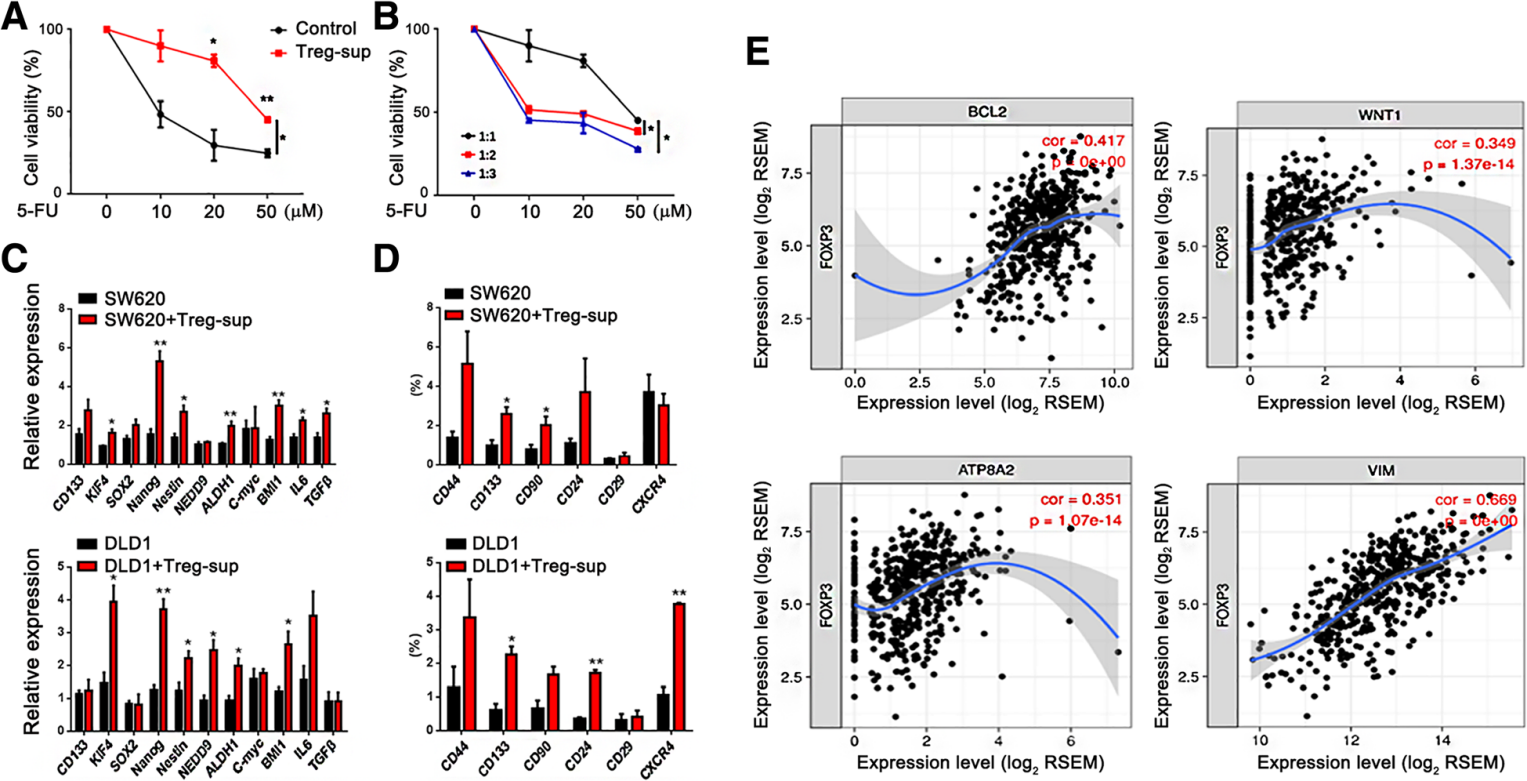
Tregs enhance the chemoresistance of CRC to 5-FU. **a** Before and after treatment with the supernatants of Tregs, cell viability of SW620 cells treated with 5-FU (10, 20, and 50 μM) was analyzed by the CCK8 assay. **b** With different concentrations of Treg supernatants (supernatant frequency of Treg/tumor cell = 1:1, 1:2 and 1:3), cell viability of SW620 cells treated with 5-FU (10, 20, and 50 μM) was analyzed by the CCK8 assay. **c** Relative expression of resistance-related genes in SW620 and DLD1 cells was analyzed by qPCR. **d** Relative expression of CSC-related genes in SW620 and DLD1 cells before and after treatment with Treg supernatants was analyzed by qPCR. **e** The relationship between FOXP3 and resistance-related genes from TCGA dataset was analyzed. *P < 0.05, **P < 0.01

Next, we evaluated the effect of Tregs on the chemoresistance of CRC cells in vitro. After treatment with the supernatants of Tregs, the proliferation of SW620 cells treated with 5-FU at different doses was significantly increased compared to the control, and in a concentration-dependent manner (Fig. 4a,b). Furthermore, the supernatants of Tregs increased the expression of resistance-related genes [22–24] in SW620 and DLD1 cells (Fig. 4c). The cancer stem cell (CSC) phenotype id one of the key characteristics of chemoresistance in tumor cells. We further investigated the expression of CSC-related genes in CRC cells after treatment with Treg supernatants. The results showed that CSC-related gene [25, 26] expression in SW620 and DLD1 cells was significantly lower than that in cells treated with Treg supernatants (Fig. 4d). We also analyzed the correlation between FOXP3 and resistance-related genes from the TCGA dataset, indicating that FOXP3 expression was closely correlated with resistance-related gene expression (Fig. 4e). All these data demonstrate that Tregs can enhance the chemoresistance of CRC cells to 5-FU.

### FOXO1/CEBPB/NF-κB signaling is required for CCL20 upregulation mediated by 5-FU

To understand the underlying mechanism of CCL20 upregulation mediated by 5-FU, we analyzed the signaling pathway correlated with CCL20 using Gene Set Enrichment Analysis (GSEA). As a result, the NF-κB signaling pathway was positively correlated with CCL20 expression (Additional file 2: Figure S2A). To verify this, the phospho-P65 level was indeed increased in SW620 cells after 5-FU treatment (Additional file 2: Figure S2B). Moreover, the levels and localization of phospho-p65, as determined by immunofluorescence, indicate that NF-κB signaling may be activated in SW620 cells by 5-FU (Additional file 2: Figure S2C). CCL20 expression in colorectal cells with or without 5-FU treatment before and after treatment with QNZ (NF-κB inhibitor) was analyzed by qPCR and ELISA. QNZ decreased the expression of CCL20 in SW620 or DLD-1 cells treated with 5-FU (Additional file 2: Figure S2D, S2E). To investigate whether P65 physically bound to the promoter region of CCL20, a dual luciferase reporter assay was performed in SW620 cells treated with or without 5-FU. The results showed that the CCL20 promoter region showed greater enrichment of P65 in SW620 cells treated with 5-FU, indicating that P65 was indeed located in the promoter region of CCL20 in SW620 cells (Additional file 2: Figure S2F). To further evaluate 5-FU-mediated Treg recruitment via NF-κB/CCL20 signaling, we investigated cell migration after NF-κB blockade by the Transwell assay and found that 5-FU-induced Treg migration could be inhibited after treatment with QNZ (Additional file 2: Figure S2G). These findings indicate that NF-κB is involved in the CCL20 expression induced by 5-FU in colorectal cells.

**Fig. 5:**
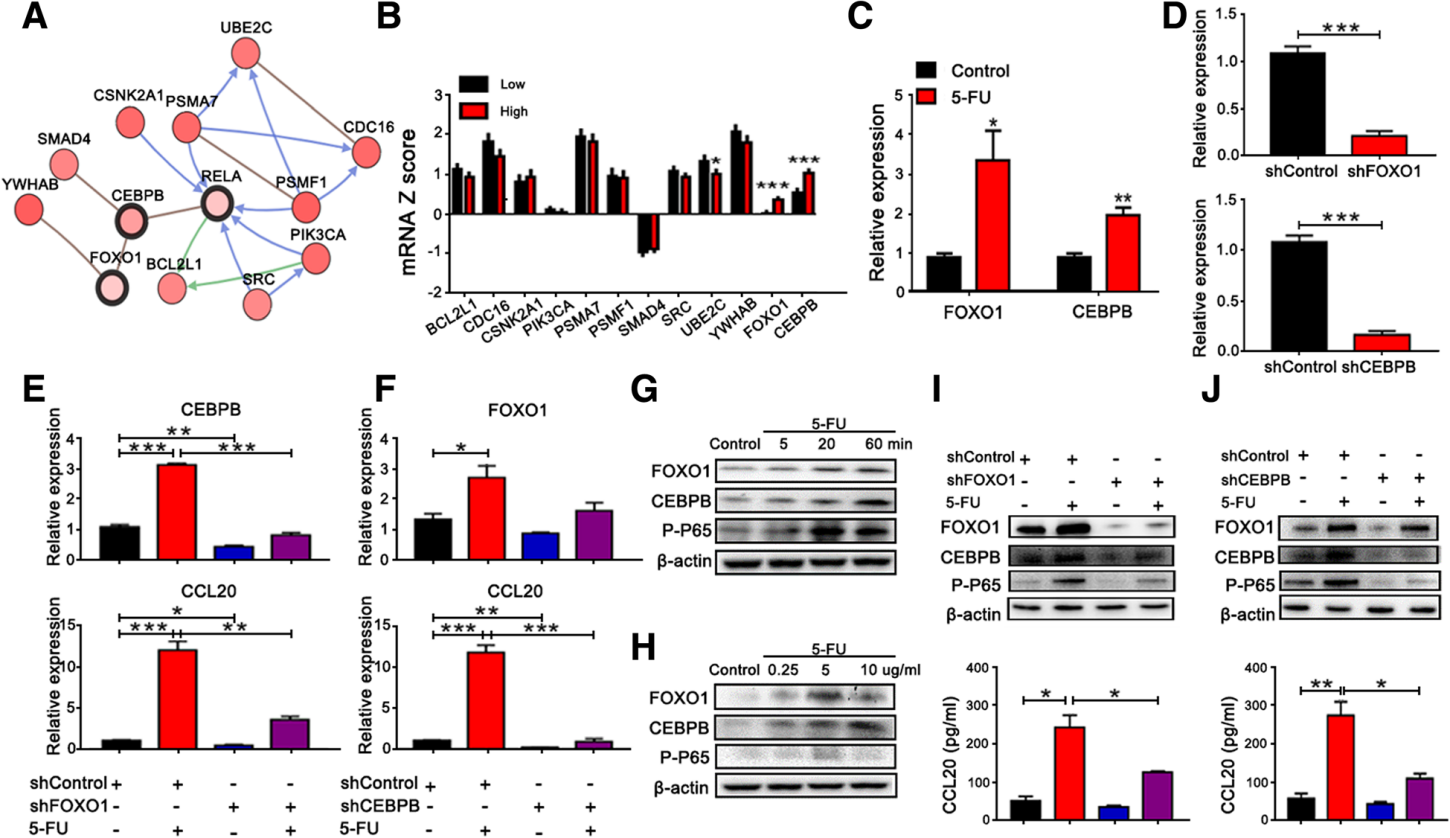
FOXO1/CEBPB/NF-κB signaling is required for CCL20 upregulation mediated by 5-FU. **a** Screening graph showing the relationship between RELA and other signaling pathways using cBioPortal for Cancer Genomics. **b** Relative expression of related genes predicted in **a** was analyzed from TCGA dataset. **c** Relative expression of FOXO1 and CEBPB in SW620 cells before and after treatment with 5-FU was analyzed by qPCR. **d** Relative expression of FOXO1 and CEBPB in stable FOXO1/CEBPB knockdown SW620 cells was analyzed by qPCR. **e** Relative expression of CEBPB and CCL20 in shFOXO1 SW620 cells treated with 5-FU was analyzed by qPCR. **f** Relative expression of FOXO1 and CCL20 in shCEBPB SW620 cells treated with 5-FU was analyzed by qPCR. FOXO1/CEBPB/NF-κB signaling activation in SW620 cells after treatment with 5-FU at different times (5, 20, and 60 min) (**g**) and with different doses (0.25, 5, and 10 μg/ml) (**h**). FOXO1, CEBPB, and phospho-P65 protein levels were assessed by western blotting. FOXO1/CEBPB/NF-κB signaling activation in shFOXO1 (**i**) and shCEBPB (**j**) SW620 cells treated with 5-FU was analyzed by western blotting. *P < 0.05, **P < 0.01, ***P < 0.001

Next, we examined which signaling pathway could regulate NF-κB/CCL20 in chemoresistant colorectal cells using the cBioportal website; we found that FOXO1/CEBPB signaling was mostly associated with RELA (NF-κB) (Fig. 5a). To verify this, the expression of the related genes predicted in Fig. 5a was analyzed from the TCGA dataset. The result showed that a high level of RELA expression was closely correlated with high levels of FOXO1 and CEBPB (Fig. 5b). After treatment with 5-FU, the expression of FOXO1 and CEBPB in SW620 cells was significantly increased compared to that in the control (Fig. 5c). To experimentally confirm that this signaling pathway is required for CCL20 upregulation mediated by 5-FU, stable FOXO1/CEBPB knockdown in SW620 cells was established using a FOXO1/CEBPB shRNA-expressing plasmid (Fig. 5d). After FOXO1 knockdown, the mRNA expression of CEBPB and CCL20 in SW620 cells treated with 5-FU was decreased significantly (Fig. 5e). Moreover, the mRNA expression of CCL20 in SW620 cells treated with 5-FU was significantly decreased after CEBPB knockdown, and no significant difference in FOXO1 was observed (Fig. 5f). Furthermore, western blotting results showed that FOXO1/CEBPB/NF-kB signaling was activated in SW620 cells after 5-FU treatment in a time- (Fig. 5g) and dose- (Fig. 5h) dependent manner. Similarly, CEBPB, phospho-P65 and CCL20 protein levels in SW620 cells treated with 5-FU were significantly decreased after FOXO1 knockdown (Fig. 5i). Phospho-P65 and CCL20 protein levels in SW620 cells treated with 5-FU were also significantly decreased after CEBPB knockdown (Fig. 5j). Accordingly, these results imply that FOXO1/CEBPB/NF-κB signaling is required for CCL20 upregulation mediated by 5-FU.

### CCL20 blockade suppresses tumor progression and restores 5-FU sensitivity in CRC

**Fig. 6:**
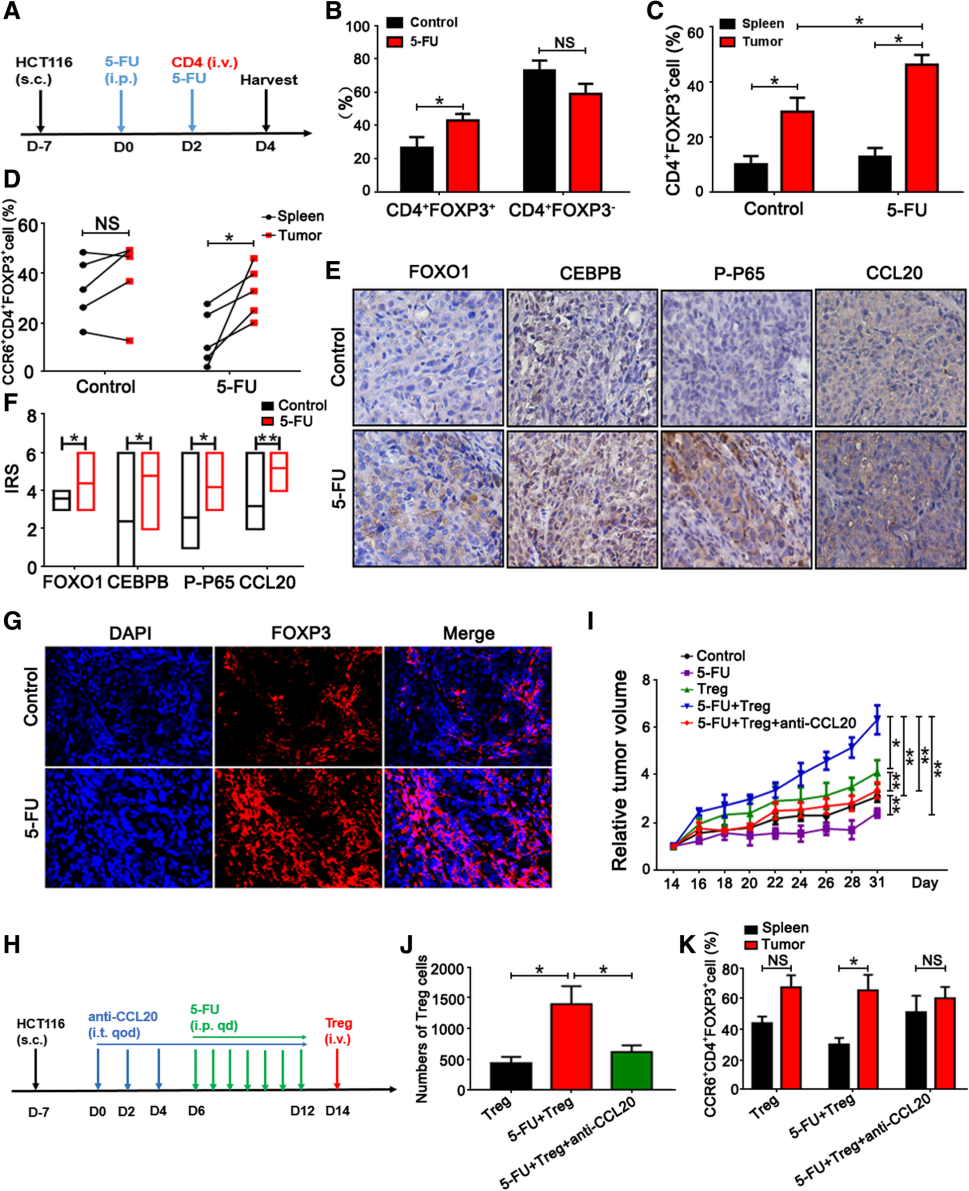
CCL20 blockade suppresses tumor progression and restores 5-FU sensitivity in CRC**. a** Graph showing the outline of HCT116 cell injection, 5-FU treatment, and CD4^+^ cell transfer in vivo. Groups received hypodermic injections of 5 × 10^6^ HCT116 cells (D-7). 5-FU (10 mg/kg/day, i.p.) treatment was started when the tumor volumes reached 250 mm^3^ (D0). Two days before the mice were sacrificed, human CD4^+^ cells (5 × 10^6^ cells) were transplanted by caudal vein (D2). **b** The percentage of CD4^+^FOXP3^+^ and CD4^+^FOXP3^−^ cells in xenografts with or without 5-FU treatment was analyzed by flow cytometry. **c** The percentage of CD4^+^FOXP3^+^ cells in xenografts and spleens with or without 5-FU treatment was analyzed by flow cytometry. **d** The percentage of CCR6^+^CD4^+^FOXP3^+^ cells in xenografts and in spleens with or without 5-FU treatment was analyzed by flow cytometry. **e** The expression of FOXO1, CEBPB, P-P65, and CCL20 in xenografts with or without 5-FU treatment was detected by immunohistochemistry (200 ×). **f** IRS of FOXO1, CEBPB, P-P65, and CCL20 in xenografts with or without 5-FU treatment as analyzed by immunohistochemistry was presented. **g** CRC tissues subjected to immunofluorescence for FOXP3 (red) and DAPI (blue). One representative micrograph is shown (200 ×). **h** Graph showing the outline of HCT116 cell injection, anti-CCL20 antibody usage, 5-FU treatment and Treg cell transfer in vivo. 5 × 10^6^ HCT116 cells were injected subcutaneously into the mice (D-7). Seven days after cell implantation, the anti-CCL20 antibody (1 mg/kg) or DMSO as a control, was administrated locally to the mice every 2 days for 2 weeks (D0, 2, 4, 6, 8, 10, 12). At day 6–12 after anti-CCL20 antibody administration, mice were treated daily with 5-FU (10 mg/kg/day, i.p.). At day 14 after anti-CCL20 antibody administration, human Tregs (5 × 10^6^ cells) were transplanted through the caudal vein (D14). After 17 days, the mice were sacrificed and tumors were isolated for further analysis. **i** Tumor volumes were measured from day 14 to day 31 after HCT116 and Treg cell implantation. The results are showed in the line chart. **j** Numbers of Treg cells in xenografts were calculated and analyzed. **k** The percentage of CCR6^+^CD4^+^FOXP3^+^ cells in xenografts and the spleens was analyzed by flow cytometry. *P < 0.05, **P < 0.01, NS- non-significant

To evaluate the in vivo function of 5-FU-mediated chemoresistance via enhancing Treg recruitment, HCT116 cells were injected subcutaneously into nude mice. When tumor volumes reached 250 mm^3^, 5-FU was injected intraperitoneally. Human CD4^+^ cells were transplanted through the caudal vein 2 days before the mice were sacrificed (Fig. 6a). The percentage of CD4^+^FOXP3^+^ cells in xenografts was increased in the 5-FU treatment group compared to that in the control (Fig. 6b). The percentage of CD4^+^FOXP3^+^ cells in xenografts treated with 5-FU was significantly higher than that in spleens also treated with 5-FU (Fig. 6c). Moreover, in the 5-FU treatment group, the CCR6^+^ Treg frequency in xenografts was obviously higher than that in the spleen (Fig. 6d). Immunohistochemistry results showed that FOXO1, CEBPB, phospho-P65, and CCL20 expression levels were increased in xenografts treated with 5-FU compared to those in the control (Fig. 6e, f). Meanwhile, FOXP3^+^ Treg infiltration was increased in xenografts treated with 5-FU (Fig. 6g). These data indicate that 5-FU enhances Treg recruitment and infiltration in colorectal tumor tissues.

To evaluate whether CCL20 blockade could restore 5-FU-mediated chemoresistance in CRC, HCT116 cells or SW620 cells were injected subcutaneously into mice (D-7). Seven days after cell implantation, the anti-CCL20 antibody was administrated locally to the mice every 2 days for 2 weeks. At day 6–12 after anti-CCL20 antibody administration, 5-FU treatment was administered to the mice daily. At day 14 after anti-CCL20 antibody usage, human Tregs were transplanted through the caudal vein (D14). Seventeen days later, the mice were sacrificed (Fig. 6h). We found that 5-FU significantly suppressed the tumor growth, which was enhanced after injecting Treg cells intravenously. However, Treg cell-mediated tumor growth was blocked by anti-CCL20 antibody, which itself had no influence on tumor growth and 5-FU-mediated effects (Fig. 6i, Additional file 3: Figure S3 and Additional file 4: Figure S4). In addition, Treg infiltration in xenografts treated with 5-FU and anti-CCL20 antibody was decreased compared to that in xenografts only treated with 5-FU (Fig. 6j). In the group with 5-FU and anti-CCL20 antibody treatment, CCR6^+^ Treg infiltration in the xenografts and spleens showed no significant difference (Fig. 6k). Collectively our results suggest that blockade of CCL20 suppresses tumor progression and restores 5-FU sensitivity in CRC, which is mediated by decreased Treg recruitment.

### Expression of signaling molecules is significantly correlated with CRC patient survival

**Fig. 7:**
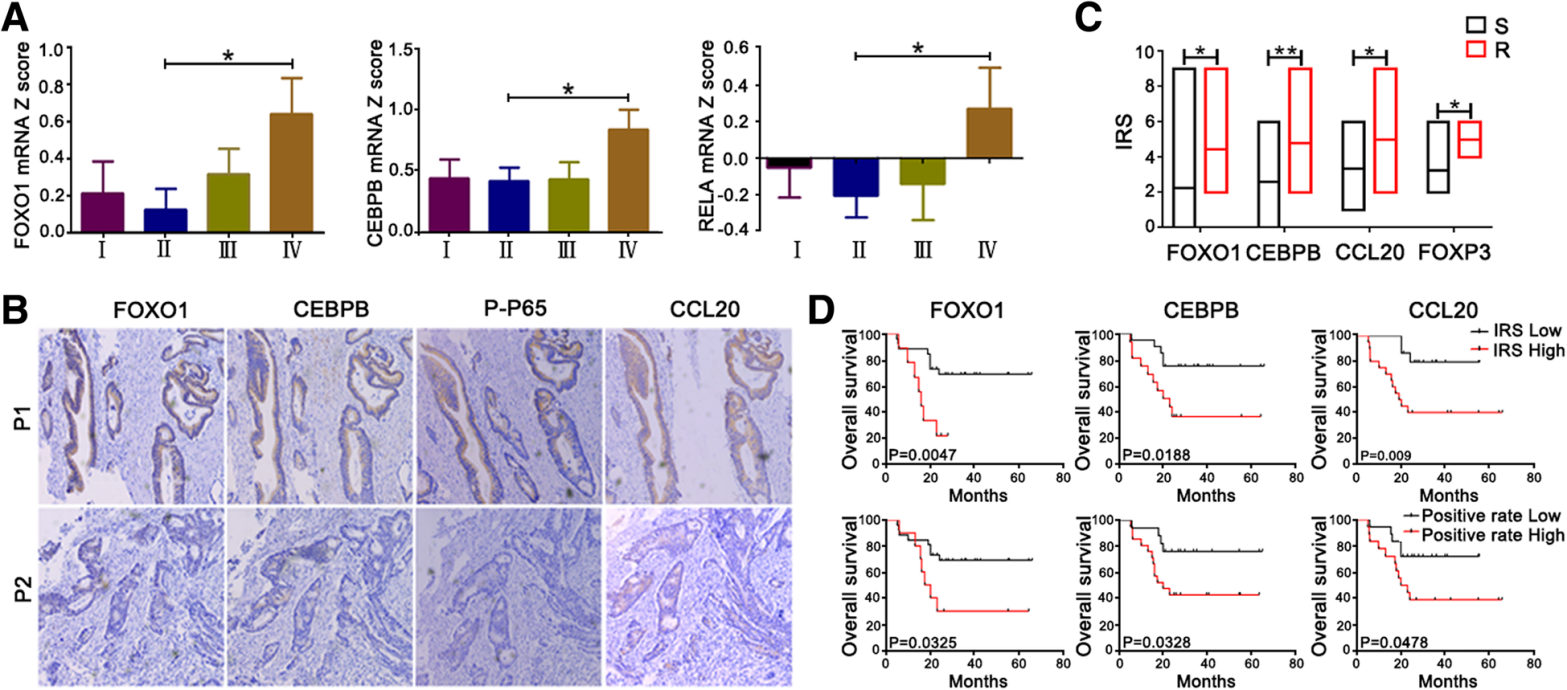
Relationship between the expression of signaling molecules and survival of CRC patients. **a** Expression of FOXO1, CEBPB, P-P65, and CCL20 in tumor tissues of CRC neoadjuvant chemotherapy patients with chemosensitivity (S) and chemoresistance (R) was detected by immunohistochemistry (100 ×). **b** IRS of FOXO1, CEBPB, CCL20, and FOXP3 in tumor tissues of CRC neoadjuvant chemotherapy patients (*n* = 55) analyzed by immunohistochemistry is presented. **c** Correlations of FOXO1, CEBPB, and RELA with CRC clinical stage were analyzed from TCGA dataset. Z-score defined as the cut-off point. **d** Kaplan-Meier survival curves for CRC neoadjuvant chemotherapy patients (*n* = 40) with lower and higher IRS or positive rate of FOXO1, CEBPB, and CCL20 expression (immunohistochemistry analysis). *P < 0.05, **P < 0.01

We next investigated whether the expression of FOXO1/CEBPB/NF-κB/CCL20 signaling molecules had prognostic value using tumor tissues from CRC patients. Firstly, the expression data of these molecules from TCGA dataset were collected and analyzed, showing that the expression of these signaling molecules was closely related (Additional file 5: Figure S5C). FOXO1, CEBPB, and RELA mRNA expression in stage IV tumor tissues was significantly higher than that in stage II tumor tissues (Fig. 7a), indicating that the signaling molecule signatures are closely correlated with the tumor stage. We further estimated the relationship between the expression of these signaling molecules. CCL20 expression was closely correlated with the expression of FOXO1 and CEBPB by qPCR (Additional file 5: Figure S5A) and immunohistochemistry (Additional file 5: Figure S5B). Moreover, CEBPB expression was closely correlated with FOXO1 (Additional file 5: Figure S5A, S5B) and P65 expression (Additional file 5: Figure S5A). FOXO1 expression was also correlated with FOXP3 expression (Additional file 5: Figure S5B). In addition, the expression of these signaling molecules in CRC patients with neoadjuvant chemotherapy was also evaluated, and we found that high levels of these molecules were present in tumor tissues from one chemoresistant patient but the levels were low in another chemosensitive patient (Fig. 7b). The data showed that FOXO1, CEBPB, and FOXP3 expression in tumor tissues from CRC patients with chemoresistance was dramatically higher than that in chemosensitive tumor tissues (Fig. 7c). Lastly, CRC patients who received neoadjuvant chemotherapy with high levels of FOXO1, CEBPB, and CCL20 in tumor tissues showed worse overall survival (Fig. 7d). Therefore, we conclude that high expression of signaling molecules is closely correlated with resistance and poor survival in CRC patients.

## Discussion

CRC is the third most common cancer with high cancer-related death worldwide [27]. Currently, recurrence and metastasis are the principal causes of death despite improvements in multidisciplinary and comprehensive treatment based on the surgical resection of CRC [28]. Despite in-depth studies on the molecular mechanisms underlying CRC for the last decades, chemoresistance remains a crucial challenge for the treatment of CRC. However, in the immunosuppressive tumor microenvironment, many factors can contribute to chemoresistance. Therefore, this study aimed to explore the molecular mechanisms of how the interaction between the tumor microenvironment regulates chemoresistance in CRC, which could provide potential targets to overcome resistance. We demonstrated the important role of CCL20 in the control of chemoresistance induced by FOXO1/CEBPB/NF-κB in CRC. Accordingly, the FOXO1/CEBPB/NF-κB/CCL20 axis might provide a potential molecular target for CRC therapy.

Chemokines play an important role in leukocyte migration [29–31]. Increasing evidences have demonstrated a close relationship between chemokine upregulation in cancer and neovascularization, tumor progression, invasion, and metastasis [32–34]. Meanwhile, a large number of experiments have reported that chemokines contribute to cancer resistance. B lymphocytes recruited by CXCL13 into the tumor site promote castration-resistant prostate cancer by producing lymphotoxin, which activates an IKKα-Bmi1 module in prostate cancer stem cells [35, 36]. Steinberg et al. observed that myeloid-derived suppressor cell (MDSC) restoration was mediated by MAPK signaling reactivation and downstream production of the myeloid attractant CCL2 in BRAFi-resistant melanoma cells. Strikingly, MDSC depletion/blockade (anti-Gr-1 + CCR2 antagonist) inhibited the outgrowth of BRAFi-resistant tumors [37]. Furthermore, Ly6Clo monocytes drive immunosuppression and confer resistance to anti-VEGFR2 cancer therapy for CRC, and CX3CR1 is critical for Ly6Clo monocyte transmigration across the endothelium in murine CRC tumors [38]. In our study, we also found a close correlation between the chemokine CCL20 and drug resistance in cancer. These results indicate that CCL20 enhanced 5-FU-resistance in CRC cells. Moreover, CRC cell-derived CCL20 promoted the recruitment of Tregs, which further induced resistance.

CCL20 is known to play an important role in tumor progression. Stromal cell-derived CCL20 promotes tumor progression and osteolysis in giant cell tumors of bone [39]. Benkheil et al. identified hepatitis C virus-induced CCL20 as a direct pro-angiogenic factor that acts on endothelial CCR6, suggesting that the CCL20/CCR6 axis contributes to hepatic angiogenesis, promoting the hypervascular state of hepatocellular carcinoma [40]. Stromal levels of CCL20 in primary melanomas may be a clinically useful marker for assessing patient risk, making treatment decisions, and planning or analyzing clinical trials [41]. In addition, CCL20/CCR6 promotes cell proliferation and metastasis in laryngeal cancer by activating the p38 pathway [42]. Stromal fibroblasts induce CCL20 through IL6/C/EBPβ to support the recruitment of Th17 cells during cervical cancer progression [43]. Benevides et al. found that IL17A induced IL6 and CCL20 production in metastatic tumor cells, favoring the recruitment and differentiation of Th17, and IL17 further promoted mammary tumor progression [44].

Our results showed that FOXO1/CEBPB/NF-κB signaling might be required for CCL20 expression to enhance chemoresistance in CRC. Nevertheless, the correlation between FOXO1/CEBPB/NF-κB and drug resistance-induced tumor progression is reported in some studies. FOXO1 is closely related with CRC progression, and also promotes invasion and metastasis of some subsets in colon and breast cancers [45]. Resistance to treatment was also ascribed to FOXO activation in multiple cases, including targeted therapies [45]. Barakat et al. demonstrate that C/EBPβ is a critical effector of autophagy via regulation of autolysosome formation and promotes resistance to proteasome inhibitor treatment by increasing autophagy [46]. Overexpression of C/EBPβ-1 increases transformation, upregulates expression of the cancer stem cell marker ALDH1A1, and leads to chemoresistance [47]. In addition, ferulic acid contributes to the reversal of multidrug resistance through suppression of P-glycoprotein expression via inhibition of the NF-κB signaling pathway [48]. A key component of inflammation-based cancer progression is elevated NF-κB activity, and in numerous cancer entities, this is associated with resistance to apoptotic cell death, promotion of cellular proliferation and an invasive and migratory phenotype [49–51].

Targeting the FOXO1/CEBPB/NF-κB/CCL20 axis in tumors may provide a novel potential therapeutic strategy for controlling CRC. MicroRNA-96 expression induced by low-dose cisplatin or doxorubicin regulates chemosensitivity, cell death, and proliferation in gastric cancer SGC7901 cells by targeting FOXO1 [52]. Piva et al. showed that functional validation of the anaplastic lymphoma kinase signature identifies CEBPB as a critical target gene [53]. Reduction of SATB2 or N-cadherin resulted in NF-κB inactivation, which led to impaired osteosarcoma sphere formation and tumor cell proliferation [54]. In the current study, we used anti-CCL20 antibody to investigate tumor growth in vivo and found that blockade of CCL20 suppressed tumor progression and restored 5-FU sensitivity in CRC, suggesting that the FOXO1/CEBPB/NF-κB/CCL20 axis may be a potential therapeutic target for CRC.

## Conclusions

In summary, high levels of CCL20 mediated the chemoresistance induced by 5-FU in CRC via FOXO1/CEBPB/NF-κB signaling. CCL20 blockade suppressed tumor progression and restored 5-FU sensitivity in CRC. Therefore, therapeutic strategies that target the FOXO1/CEBPB/NF-κB/CCL20 axis could represent an effective method for CRC treatment.

## Additional files

Additional file 1: **Figure S1.** CCL20 level is increased in the serum of chemoresistant patients. (TIF 3387 kb)

Additional file 2: **Figure S2.** NF-κB is involved in CCL20 expression induced by 5-FU in colorectal cells. (TIF 3281 kb)

Additional file 3: **Figure S3.** Treatment with anti-CCL20 antibody had no influence on tumor growth and 5-FU-mediated effects. (TIF 2189 kb)

Additional file 4: **Figure S4.** CCL20 blockade suppresses tumor progression and restores 5-FU sensitivity in SW620 cells. (JPG 17 kb)

Additional file 5: **Figure S5.** The relationship between the signaling molecule expressions. (TIF 5404 kb)

## Data Availability

The datasets used and/or analyzed during the current study are available from the corresponding author on reasonable request.

## Abbreviations

CCL20: Chemokine (C-C motif) ligand 20
CRC: Colorectal cancer
CSC: Cancer stem cell
GFP: Green fluorescent protein
GO: Gene Ontology
GSEA: Gene Set Enrichment Analysis
MDSC: Myeloid-derived suppressor cell
PBMCs: Peripheral blood mononuclear cells
TCGA: The Cancer Genome Atlas
TILs: Tumor infiltrating lymphocytes
Treg: Regulatory T cells
