# Supplementary figures and images for: Colorectal cancer cell-derived CCL20 recruits regulatory T cells to promote chemoresistance via FOXO1/CEBPB/NF-κB signaling
Source: J Immunother Cancer. 2019 Aug 8;7:215. doi: 10.1186/s40425-019-0701-2 (PMC6688336; doi:10.1186/s40425-019-0701-2)

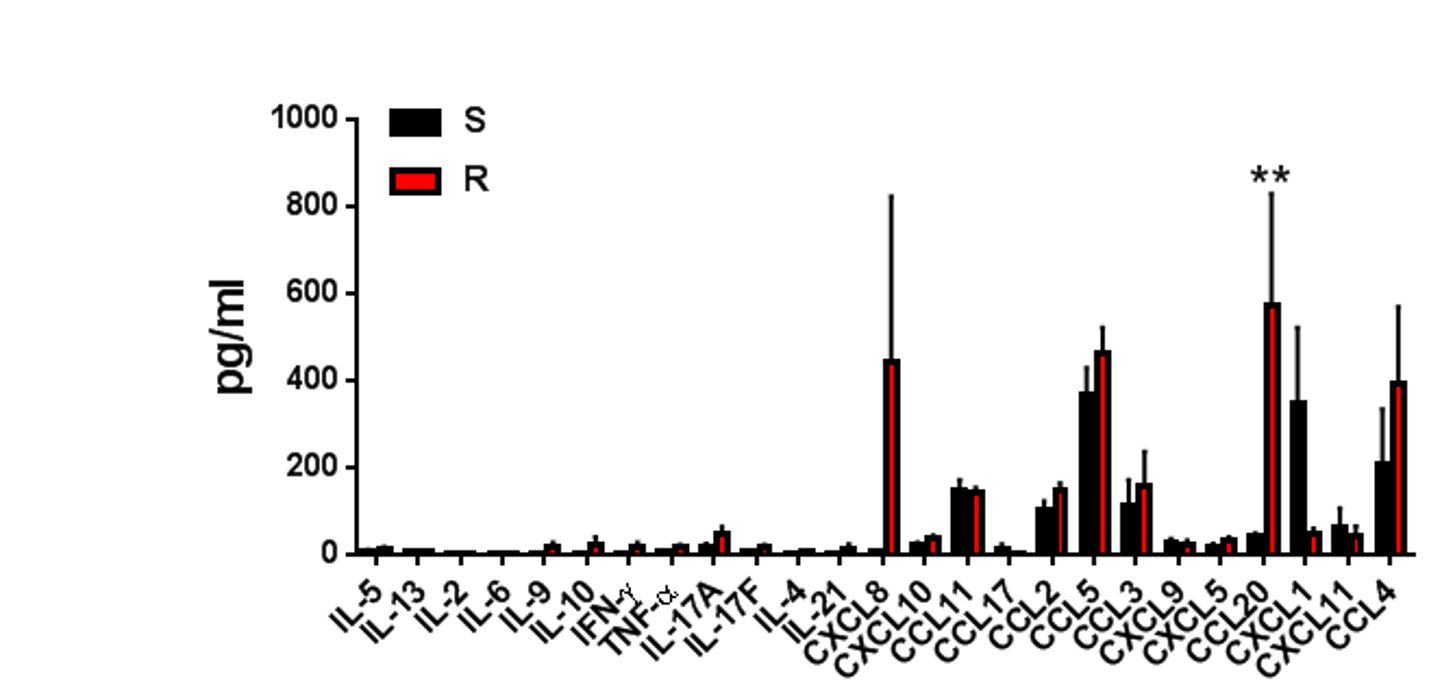

Supplement: Supplementary file 1 — Figure S1. CCL20 level is increased in the serum of chemoresistant patients. (TIF 3387 kb) [file 40425_2019_701_MOESM1_ESM.tif]

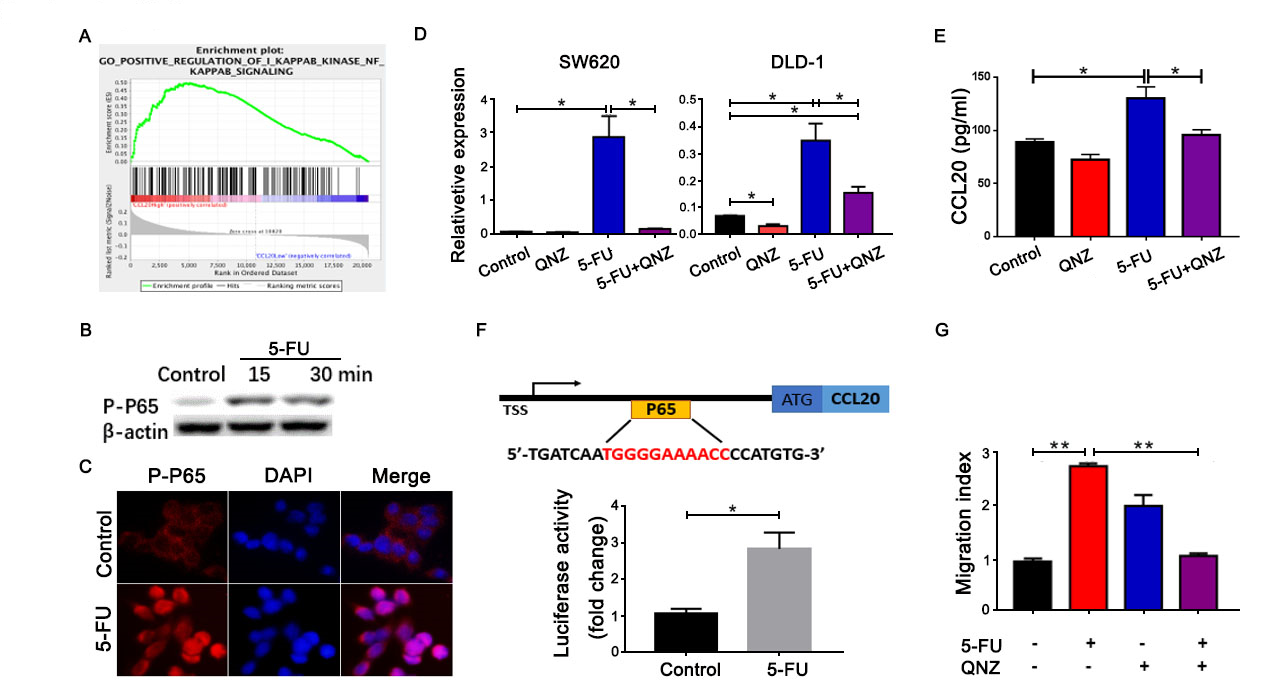

Supplement: Supplementary file 2 — Figure S2. NF-κB is involved in CCL20 expression induced by 5-FU in colorectal cells. (TIF 3281 kb) [file 40425_2019_701_MOESM2_ESM.tif]

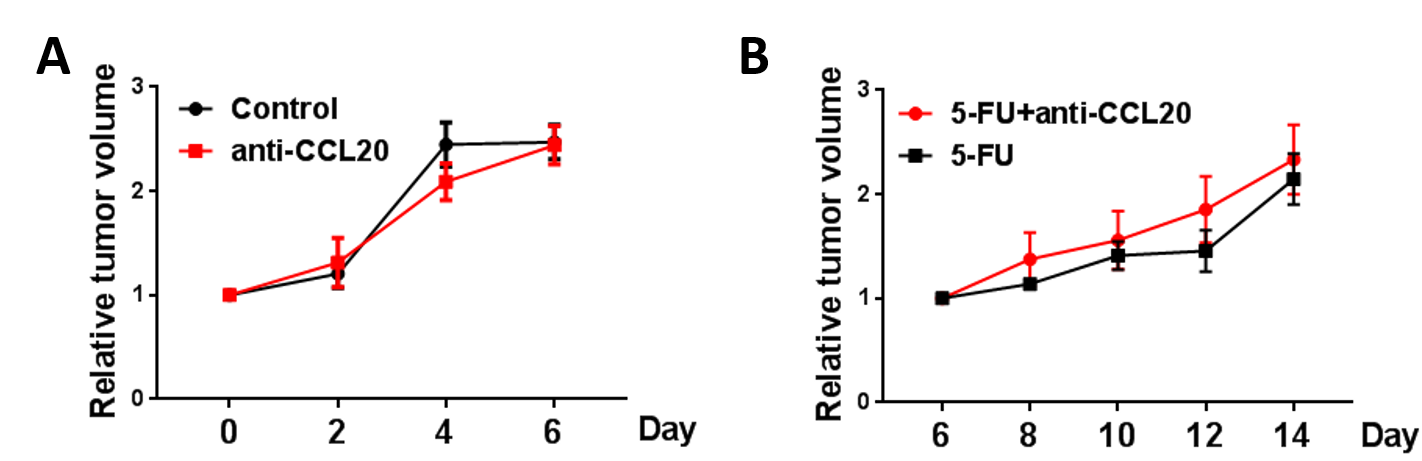

Supplement: Supplementary file 3 — Figure S3. Treatment with anti-CCL20 antibody had no influence on tumor growth and 5-FU-mediated effects. (TIF 2189 kb) [file 40425_2019_701_MOESM3_ESM.tif]

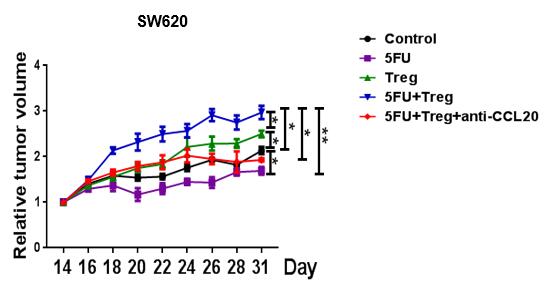

Supplement: Supplementary file 4 — Figure S4. CCL20 blockade suppresses tumor progression and restores 5-FU sensitivity in SW620 cells. (JPG 17 kb) [file 40425_2019_701_MOESM4_ESM.jpg]

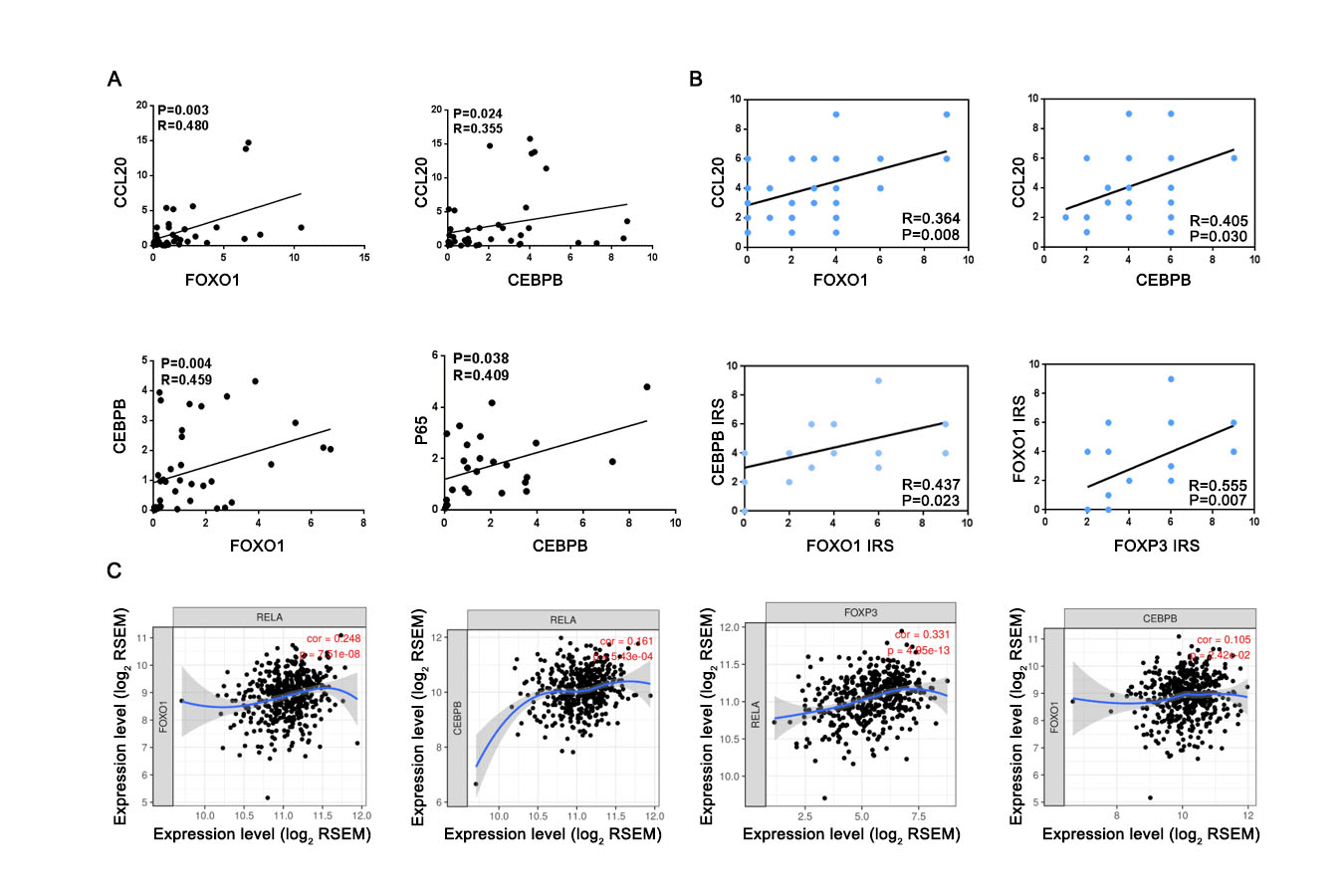

Supplement: Supplementary file 5 — Figure S5. The relationship between the signaling molecule expressions. (TIF 5404 kb) [file 40425_2019_701_MOESM5_ESM.tif]
